# Concurrent Validity of Cervical Movement Tests Using VR Technology—Taking the Lab to the Clinic

**DOI:** 10.3390/s23249864

**Published:** 2023-12-16

**Authors:** Karin Forsberg, Johan Jirlén, Inger Jacobson, Ulrik Röijezon

**Affiliations:** Department of Health, Education, and Technology, Luleå University of Technology, 97187 Luleå, Sweden; johan.jirlen@ltu.se (J.J.); inger.jacobson@ltu.se (I.J.); ulrik.roijezon@ltu.se (U.R.)

**Keywords:** neck pain, cervical, virtual reality, VR, 3D motion capture, validity, agreement, correlation, range of motion, velocity

## Abstract

Reduced cervical range of motion (ROM) and movement velocity are often seen in people with neck pain. Objective assessment of movement characteristics is important to identify dysfunction, to inform tailored interventions, and for the evaluation of the treatment effect. The purpose of this study was to investigate the concurrent validity of a newly developed VR technology for the assessment of cervical ROM and movement velocity. VR technology was compared against a gold-standard three-dimensional optical motion capture system. Consequently, 20 people, 13 without and 7 with neck pain, participated in this quantitative cross-sectional study. ROM was assessed according to right/left rotation, flexion, extension, right/left lateral flexion, and four diagonal directions. Velocity was assessed according to fast cervical rotation to the right and left. The correlations between VR and the optical system for cervical ROM and velocity were excellent, with intraclass correlation coefficient (ICC) values > 0.95. The mean biases between VR and the optical system were ≤ 2.1° for the ROM variables, <12°/s for maximum velocity, and ≤3.0°/s for mean velocity. In conclusion, VR is a useful assessment device for ROM and velocity measurements with clinically acceptable biases. It is a feasible tool for the objective measurement of cervical kinematics in the clinic.

## 1. Introduction

Neck pain is one of the most common musculoskeletal conditions globally [1], with a point prevalence of 3.6% [2], and a one-year prevalence of 30–50% [3]. Musculoskeletal disorders, including neck pain, are common causes for sick leave [4], causing high economic costs [2,5,6]. Reduced cervical range of motion (ROM) [7,8,9,10,11] and movement velocity [12,13,14,15,16,17] are clinical signs which often present in people with neck pain. Peak velocity clearly differs between people with and without neck pain, with high sensitivity and specificity [12,15].

Testing cervical ROM is a core assessment in patients with neck pain [8,18]. The ability to move our head in different directions is vital for daily functioning and activities, for example, driving a car, riding a bike, or walking across the street while watching out for traffic. Clinical assessment of ROM is commonly performed using analog methods such as goniometers, inclinometers, visual estimation, or tape measurements, with varying accuracy [19]. In research and some specialized clinics, specific laboratory equipment is used, e.g., optoelectronic camera systems [20,21], electromagnetic systems [15], ultrasound systems [22], or inertial measurement units (IMUs) [23]. For standardization, cervical ROM is traditionally measured in each of the sagittal, frontal, and transverse movement planes. However, functional movement often combines several movement planes, e.g., diagonal movements to follow a visual target. Objective assessment of the maximum ROM in diagonal motion may have a clinical value but has, to our knowledge, not been investigated. New affordable technology, such as virtual reality (VR) and other IMU systems, enables feasible, accurate, and automatized measurements of ROM in the clinic [11,23,24,25].

Assessments of cervical movement velocity are rarely performed in the clinic, as movement velocity cannot be measured using the traditional analog methods. Movement velocity is usually assessed in laboratory settings using more complex systems [15,16,26]. More recently, new affordable technology has become available for clinical use, e.g., VR glasses [12,27].

VR technology is a novel, promising method with the potential to assess kinematics similarly to more advanced laboratory equipment. VR technology has the potential to give objective assessments of important neck functions such as ROM and velocity, to guide a targeted, precise intervention, and to be used as a follow-up evaluation tool. For them to be useful in clinical and research settings, it is important to know the validity of new technologies [28,29]. This includes both the hardware and the software used in the assessment of movement functions. VR technologies for the assessment of cervical ROM and cervical movement velocity have shown good discriminant validity in identifying people with and without neck pain [11,12]. Their concurrent validity has been investigated. The accuracy of the head-mounted device Oculus Rift VR was tested against the Optotrak motion tracking reference system for cervical ROM. The Oculus Rift was found to be valid for the full excursion of movement in each of the sagittal, transverse, and frontal planes. It was less accurate when movement was measured from a neutral position to the end of range in each movement direction. VR measures differed on average ± 5°, and there were large variations between subjects [21]. In another study, head movement was tracked using two VR systems, Oculus VR and HTC Vive, and compared to the Qualisys optical motion capture system. The results showed high and moderate correlations between measures [30]. Thus, the validity of measuring range of movement using VR technology is promising, but to date, the research is lacking on the concurrent validity of the measurement of cervical ROM in diagonal movements and movement velocity.

This study evaluated the concurrent validity of head-mounted display VR technology for the assessment of ROM around the traditional vertical, frontal, and sagittal axes, as well as in diagonal movements involving all axes. We also investigated the movement velocity in fast cervical rotation using the same technology. The primary objective was to evaluate the concurrent validity of measuring the cervical ROM and velocity using VR glasses compared with a gold-standard optical motion capture system. A secondary objective was to evaluate VR’s repeatability of these tests.

## 2. Materials and Methods

A cross-sectional concurrent validity design was used to study VR-based cervical movement tests as compared to using a gold-standard optical motion capture system. Written consent was received from each participant and an ethical clearance was gained from the Swedish Ethical Review Authority (ref No: 2022-00183-01).

The participants were recruited as a convenience sample, via e-mail advertisements to staff and students at Luleå University of Technology, Sweden. People responding to the advertisement were contacted and screened for eligible criteria. The inclusion criteria were being 18–65 years of age and able to read and write in Swedish. The participants could be either non-symptomatic or have neck pain. The aim was to have a mix of neck status to test the VR technology on a variety of movement ranges and velocities. The aim was not to investigate group differences between people with and without neck pain. Participants were analyzed as one group. The exclusion criteria were neck fracture, neck surgery, cervical radiculopathy, neurological disease, epilepsy, uncorrected impaired vision, and previous experience of severe symptoms (nausea/dizziness) using VR glasses. People meeting the inclusion criteria were, after signing informed consent, enrolled in the study.

The sample size was determined in a power analysis where an ICC of 0.9 was statistically significantly higher than a set a null hypothesis of ICC 0.6, with *p* < 0.05, and 95% power. This analysis determined that 20 participants were required [30].

### 2.1. Measurement Devices

**Figure 1:**
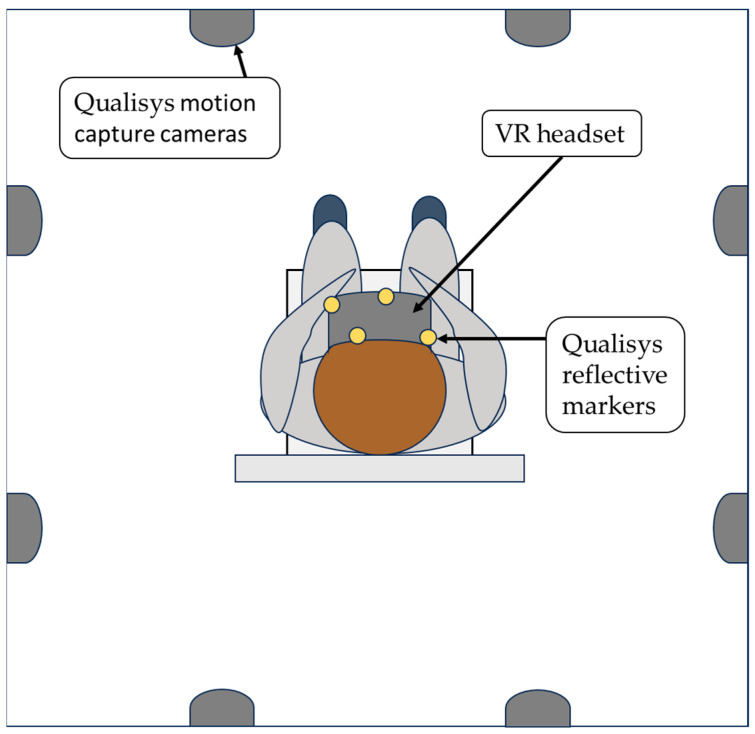
Test setup in the movement laboratory, viewed from above. Illustrative figure, scaling not accurate. Yellow dots represent Qualisys reflective markers attached to the VR glasses. As test participants moved their heads, kinematics from the VR technology and Qualisys were obtained simultaneously.

The cervical movement tests were assessed using immersive head-mounted VR-glasses, with software CurestVR_LTU-20221003 from Curest AB, (Luleå, Sweden), and the hardware Pico G2 4K (Pico Technology Co., Ltd., Cambridgeshire, UK). Immersive VR refers to the 3D virtual environment being displayed in the VR glasses and experienced by the user [31]. The VR glasses have a built-in IMU sensor, making it possible to track movements in 3 degrees of freedom (DoF), around the *x-, y*-, and *z*-axes, with a sampling rate of up to 100 Hz. The sensor uses a gyroscope, accelerometer, and magnetometer to track movements. Data are collected in real time via a portal. The VR screen has a resolution of 3849 × 2160 pixels, and the luminosity/brightness can be adjusted. The display has a built-in eye-protective blue-light-reducing system. The weight of the VR goggles is 276 g and they have adjustable straps for individual comfort. The participants performed the tests by moving their head as instructed on the screen. Before each test session, a geomagnetic calibration of the VR technology is performed, as is a calibration in the transverse plane. The sagittal position is automatically set to correspond to the horizontal level in the room. Measures with the VR and the Qualisys optoelectronic motion capture system (Qualisys AB, Göteborg, Sweden) were taken simultaneously, enabling comparison between the two systems. Qualisys uses reflective markers attached to the moving object, which are detected using cameras with high accuracy [32]. Qualisys is regarded a gold-standard system for three-dimensional motion capture [30,33]. Four reflective markers were placed on the VR goggles to enable the Qualisys system to track the cervical movements (Figure 1).

### 2.2. Data Collection

The cervical movement test protocol consisted of ROM and fast cervical rotations. The tests were performed at the Human Health and Performance Lab—Movement Science at Luleå University of Technology, Sweden, during autumn 2022. An experienced registered physiotherapist (K.F.) gave all test instructions and supervised the test performance. A laboratory technician assisted with the data collection (J.J.). All participants completed a background questionnaire, including personal questions and questions about neck status, physical activity, and function. The intensity of any neck pain was rated on the Numeric Rating Scale (NRS), a valid and reliable pain assessment tool [34].

The participant sat on a chair, 45 cm in height, with both feet on the ground and their back against the backrest (Figure 1). Each participant was instructed to sit in an erect neutral position. The back being against the backrest was to minimize upper and lower trunk movements and the participant was instructed to only move their head in the test and hold the trunk still. A standardized warmup program (without VR glasses) was conducted. Participants were taught and practiced one repetition of each test movement included in the study. Furthermore, for familiarization, each participant performed one practice trial in the VR environment before each test measurement.

**Figure 2:**
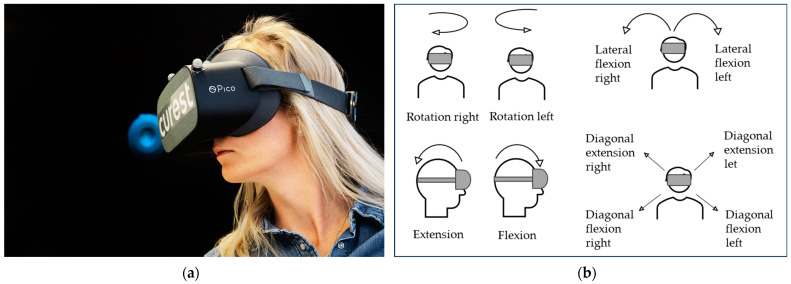
VR range of motion (ROM) test. (**a**) The black background with the blue disk shows the screen participants see in the VR glasses. The participant moves the disk from neutral position to maximum ROM in each movement direction. (**b**) Each movement direction performed in the VR-based ROM test.

In the ROM test (Figure 2), participants were instructed to move a disk on the VR screen with their head as far as possible in right rotation, left rotation, extension, flexion, left diagonal extension, right diagonal flexion, left diagonal flexion, right diagonal extension, left lateral flexion, and right lateral flexion. All directions were performed twice in this sequence. The maximum range of motion in each direction in degrees (°) was used for the data analysis. The first repetition served as the outcome variable for correlation and agreement analyses between the measurement systems, VR and Qualisys. The first and second repetitions were compared in the repeatability analysis for the VR measurement system.

Velocity in fast cervical rotations was assessed with participants performing three repetitions of cervical rotation as fast as possible to the right and left. The mean peak and mean velocity in degrees per seconds (°/s) from the 3 repetitions for each direction were used for the data analysis between the VR and Qualisys. For repeatability, data from VR’s first and second repetitions in right and left rotation, respectively, were used.

### 2.3. Calculation of Outcome Variables

**Figure 3:**
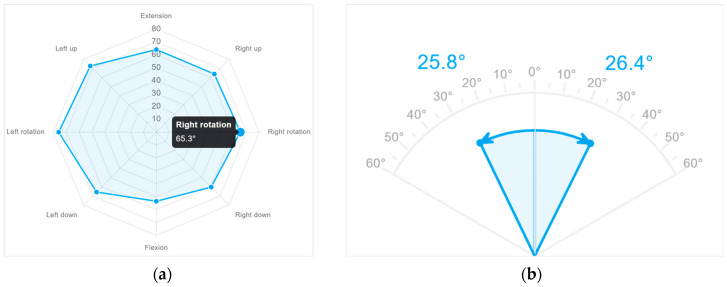
Example of ROM results displayed in the online VR portal. The maximum value for each movement direction and each repetition was exported. (**a**) ROM in rotation right, rotation left, extension, flexion, and four diagonal directions; (**b**) lateral flexion right and lateral flexion left.

The motion data from Qualisys were collected using the software program Qualisys Track Manager (QTM), version 2021.2, (Göteborg, Sweden). Prior to the ROM and velocity tests, the participant sat in their neutral head position and the VR screen was calibrated according to the transverse plane. At the same time, a time stamp was made in QTM to match temporally the VR and Qualisys systems. The origin of the VR ROM tests is determined from the disk position at the start of the test. The VR software automatically generates the maximum range of motion values for each movement direction in an online portal (Figure 3). These values were exported and used as the VR ROM variables.

The programming platform Matlab, version R2023a, with Phased Array System Toolbox, was used for the calculation of the ROM variables from QTM. Gyroscope data from VR were exported as Euler angles in pitch (sagittal, *y*), yaw (transversal, *z*), and roll (frontal, *x*) order (*yzx*). To adhere to how the portal values were calculated, they were first transformed into a vector in 3D space with *x* corresponding to the forward-facing direction of the headset. Rotational data from QTM were exported as rotational matrices for a rigid body formed from the trackers on the headset. The matrices were used to rotate a forward-pointing (*x*-direction) vector to obtain a vector representing the headset data. Angles of extension were calculated from the vector to the starting axis.

$$\theta_{right\;left}=\tan^{-1}\frac{y}{\sqrt{x^{2}+z^{2}}}$$

$$\theta_{up\;down}=\tan^{-1}\frac{z}{\sqrt{x^{2}+y^{2}}}$$

$$\theta_{diagonal}=\tan^{-1}\frac{\sqrt{y^{2}+z^{2}}}{x}$$

The headset with the current software did not align the pitch (sagittal) of the marker with the forward direction of the headset. Hence, the QTM data were shifted in pitch to align the measurements by rotating all tracked rotational positions using a matrix generated from the difference in angle at a known time using the Matlab function “roty”. The time for defining the pitch origin was calculated from the peak in the yaw when the participant returned to the starting position (VR origin) after performing the designated movement.

The rotational velocity variables were calculated in Matlab, from the measured range in rotation divided by the time. For QTM, the frequency was set to 100 Hz. The frequency of the VR was obtained from the difference between datapoint timestamps. The window for the average velocity was defined as beginning when the velocity reached 5% of the maximum velocity for the repetition, and ending when decreasing below 5%. The VR velocity was smoothed using a moving average five datapoints wide to avoid noise disturbing the initiation and end of the window and the noise spike at maximum velocity. The QTM data were not smoothed.

### 2.4. Statistics

All data variables were imported into the software program Statistical Package of Social Sciences (SPSS), version 28.0.0.0 (190) (IBM, Armonk, NY, USA). All the data variables were first checked for normality using the Shapiro–Wilk test, histograms, skewness, and kurtosis. A paired-samples *t*-test was performed to analyze the difference between the VR data and the Qualisys data, with a significance level of <0.05. The correlation between the VR data and Qualisys data was analyzed using scatter plots and Pearson’s correlation coefficient r, where values close to −1 or 1 indicate strong linear relationship. Pearson’s r values were interpreted as 0.00–0.10 = negligible correlation, 0.1–0.39 = weak correlation, 0.4–0.69 = moderate correlation, 0.7–0.89 = strong correlation, and 0.9–1.00 = very strong correlation [35]. An intraclass correlation coefficient (ICC) of 2.1, two-way random effects, and absolute agreement were also used to analyze the correlation between the two measurements, where values < 0.5 were poor, 0.5–0.75 moderate, 0.75–0.9 good, and >0.90 excellent [36]. The Bland–Altman method was used to analyze the agreement between measures. The method uses the differences between the two systems (Qualisys motion tracking minus VR) plotted against the mean of both measures. The mean difference is presented together with 95% limits of agreement (LOA), which is the mean difference ± 1.96 × standard deviation [37,38]. When the difference variables were not normally distributed, a non-parametric Bland–Altman approach was used, using the median bias and 5th–90th or 95th percentile as the limits of agreement [39]. The repeatability between the first and second repetitions of the ROM and velocity VR data was analyzed using the ICC (3.1), two-way mixed effects, absolute agreement [36], Pearson’s r, paired-samples *t*-test, Bland–Altman mean difference, and 95% LOA.

## 3. Results

All 20 participants (10 women and 10 men) performed the tests successfully. The mean age was 45 (±12) years, ranging from 21 to 62 years, weight 82 (±17) kg, and height 174 (±10) cm. Equally, 13 of 20 participants were asymptomatic, i.e., no neck pain. Seven participants reported current neck pain, with a numeric rating scale median of 2/10 (IQR 2), range 1–4. The mean duration of neck pain from the seven participants reporting neck pain was 5.7 (±5.6) years, ranging 2 weeks to 14 years. Velocity data from the VR for one participant failed to export and were excluded from the analysis, leaving n = 19 for the velocity analysis.

The results for Bland–Altman analysis are presented with mean bias and 95% LOA for all variables for simplicity and readability. However, as some of the values were not normally distributed, the median bias and percentiles are presented in tables and plots for the non-normally distributed variables.

### 3.1. Range of Motion

The mean ROM values from VR and Qualisys are presented in Table 1 together with the mean differences and *t*-test analyses. The paired-samples *t*-test showed a significant difference between VR and Qualisys for right rotation, with a mean difference = 2.1° (standard deviation 1.5°). No other ROM variables showed significant differences between VR and Qualisys.

#### 3.1.1. Correlation between VR and Qualisys for ROM Variables

Pearson’s correlation coefficient from the analysis between VR and Qualisys showed values > 0.96 (*p* < 0.001) for all directions of cervical motion, indicating very strong correlations (Table 2). The ICC values for the correlations between the VR and Qualisys ROM were significant (*p* < 0.001), and all ICC values were above 0.95 (Table 2), indicating an excellent correlation.

#### 3.1.2. Agreement between VR and Qualisys Motion Capture ROM

**Figure 4:**
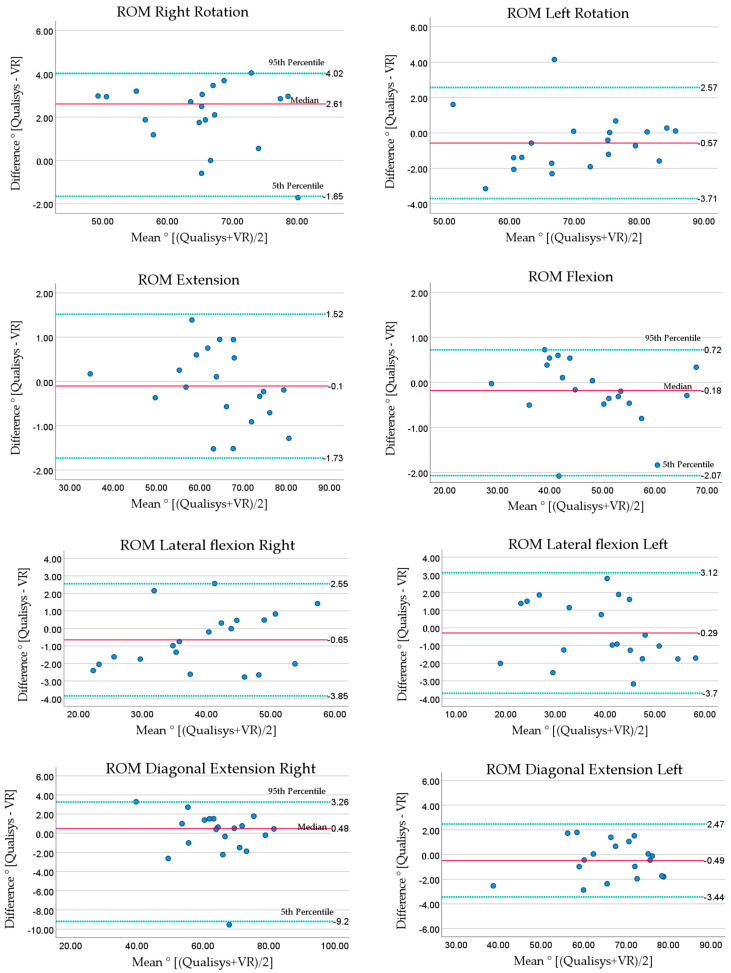

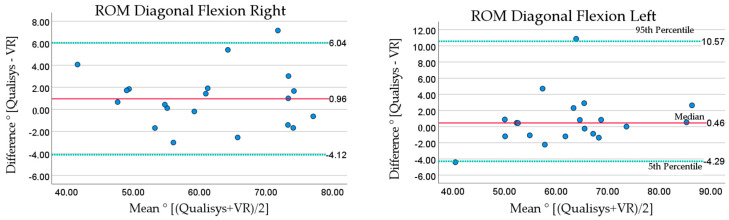
Bland–Altman plots for ROM. Qualisys compared to VR. Solid line (red) shows mean or median bias, dotted lines (green) show lower and upper limits of agreement (median and percentiles are labeled). Lower LOA = mean − (1.96 × standard deviation), upper LOA = mean + (1.96 × standard deviation). Medians are presented with 5th and 95th percentiles.

**Figure 5:**
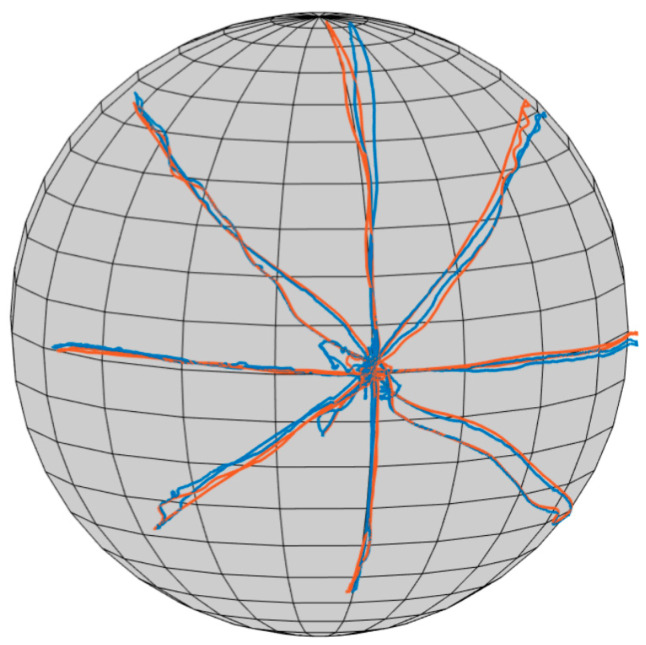
Example of a participant’s ROM tracking from VR and Qualisys. Tracking in cervical rotation right, rotation left, extension, flexion, and four diagonal directions. Blue: Qualisys. Red: VR.

The mean biases between VR and Qualisys (Table 3 and Figure 4) were ≤1° (absolute value) for 9 of 10 ROM directions. The exception was 2.1° for rotation right. Larger LOAs were found for the diagonal movements, compared to the conventional movement directions. The LOA were ≤±3.4° for the conventional movements and ≤±6.1° for the diagonal movements. The tracking data from VR and Qualisys showed signals close to each other, indicating good agreement (Figure 5).

### 3.2. Velocity

The mean values for the velocity variables from Qualisys and VR, mean differences, and *t*-test analyses are presented in Table 4. The analysis revealed no significant differences between VR and Qualisys for any of the velocity variables (Table 4).

#### 3.2.1. Correlation between VR and Qualisys Velocity Measurements

For the movement velocity tests, Pearson’s correlation and ICC analysis revealed values > 0.97 (*p* < 0.001), indicating very strong and excellent correlations between VR and Qualisys (Table 5).

#### 3.2.2. Agreement between VR and Qualisys Velocity Measures

**Figure 6:**
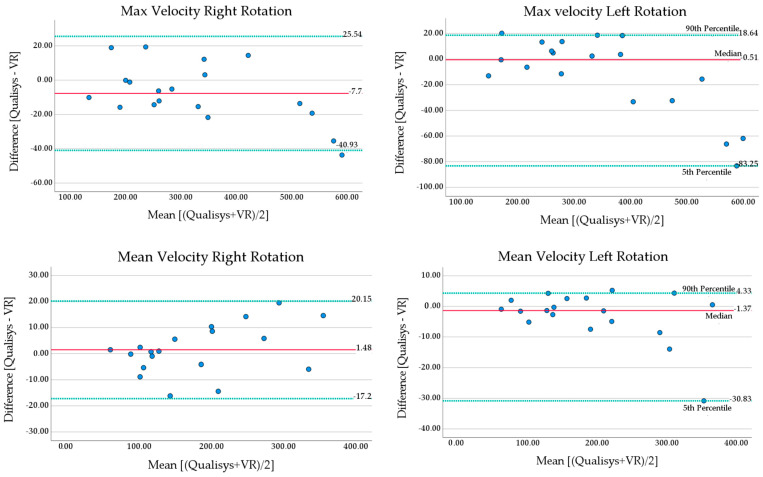
Bland–Altman plots maximum and mean velocity in fast cervical rotations. Solid line (red) shows mean or median bias, dotted lines (green) show lower and upper limits of agreement (median and percentiles are labeled). Lower LOA = mean − (1.96 × standard deviation), upper LOA = mean + (1.96 × standard deviation). Medians are presented with 5th and 90th percentiles.

Bland–Altman analysis of the velocity (Table 6 and Figure 6) revealed absolute mean differences < 12°/s for maximum velocity, with a mean difference ≤ 3.0°/s for mean velocity. The LOA for maximum velocity were less than ± 60°/s. Bland–Altman plots (Figure 6) showed that the largest differences for maximum velocity occurred at very high velocities, above 550°/s. The LOA for mean velocity were less than ±18.7°/s.

### 3.3. Repeatability of VR’s Range of Motion Variables

The repeatability for the ROM variables was calculated from the VR’s first and second repetitions. The tables for repeatability are presented in Appendix A.

The mean values for the first and second repetitions from the VR, mean differences, and *t*-test analyses are presented in Table A1. The analysis revealed a significant difference between repetitions 1 and 2 for lateral flexion left, with a mean difference = 1.3 (±2.6)°, *p* = 0.034. No other ROM directions showed significant difference between repetitions 1 and 2.

#### 3.3.1. Correlation of VR’s ROM Repetitions 1 and 2

In the correlation analysis between the VR’s ROM repetitions 1 and 2, both Pearson’s r and the ICC showed values > 0.85 (*p* < 0.001) for flexion and diagonal extension right, indicating good and strong correlations. The other eight variables showed values > 0.9 (*p* < 0.001) for the repeated measurements, indicating very strong and excellent correlations (Table A2).

#### 3.3.2. Agreement of VR’s ROM Repetitions 1 and 2

The agreement between the VR’s first and second ROM measurements are presented with mean and median biases and their LOA (Table A3). The absolute mean biases were ≤1.3° between repetitions 1 and 2 for the ROM variables. The highest LOA were ±11.4°, indicating widespread differences between participants.

### 3.4. Repeatability of VR Velocity

The repeatability of the cervical movement velocity was calculated from the VR’s first and second repetitions in right and left rotation. The mean values for VR’s repetitions 1 and 2, the mean differences, and *t*-test analyses are presented in Table A4. No significant difference between the first and second repetitions was found for the velocity variables.

#### 3.4.1. Correlation of VR’s Velocity Repetitions 1 and 2

Pearson’s correlation coefficients for the VR’s first and second repetitions in the velocity test were >0.84 (*p* < 0.001), indicating strong correlation. The ICC values were >0.8 (*p* < 0.001), indicating good correlation (Table A5).

#### 3.4.2. Agreement of VR’s Velocity Repetitions 1 and 2

The agreement between the VR’s first and second velocity repetitions in right and left rotation are presented with mean and median biases and their LOA (Table A6). The absolute mean biases were <32°/s between repetitions 1 and 2 for the velocity variables. Both the maximum and mean velocity variables had very large LOA (largest LOA ± 162°/s), indicating widespread mean biases between participants.

## 4. Discussion

This study investigated the concurrent validity of a newly developed VR technology for the assessment of cervical range of motion and cervical movement velocity in a cohort of people with and without neck pain. The measures included cervical ROM in the diagonal plane, which was a novel aspect of the study. The ICC values were >0.95 for the VR technology compared to the gold-standard Qualisys motion capture system for all ROM and velocity variables. The mean biases between VR and Qualisys were ≤2.1° (LOA ≤ ±6.1°) for the cervical ROM variables, ≤3.0°/s (LOA < ±18.7°/s) for the mean velocity, and < 12°/s (LOA < ±60°/s) for the maximum velocity variables. The mean biases were considered small, seen in relation to mean ROM values of 40–70° and mean velocities of 180–355°/s. These results indicate the good concurrent validity of the VR technology. This is of important clinical value as the VR-based cervical movement tests provide valid measures which can guide a specific individualized tailored intervention plan with targeted treatment.

This cohort’s cervical ROM values were slightly lower compared to the reported values for asymptomatic people [8]. Our group had seven participants with neck pain, who contributed to the lower mean ROM values. The mean values from the 13 asymptomatic participants were within other reported ROM normal values [8], although the values for right rotation 65.1° and flexion 49.9° were at the lower end of the normative range (rotation right 66–79°, flexion 47–63° [8]). These small values in flexion could partly be related to the sagittal starting position definition in the VR system, which is different from the participants’ self-chosen sagittal starting positions. Recommendations to the Curest VR software have been made to change the neutral calibration for all movement planes to better represent the person’s actual neutral neck position. The systematic mean bias in right rotation in our study, where VR gave 2.1° (SD 1,5°) lower values compared to Qualisys, could partly explain our lower values in right rotation compared to other studies. Regarding the bias in right rotation, we could not find any explanation for this systematic bias in our data or algorithms. However, a study evaluating Oculus Rift (Irvine, CA, USA) found an artificial drift at the beginning of the cervical movement test of about 6°, which is likely to be compensation for the drift of the IMU sensor during the measurements [21]. Since rotation right is the first movement direction in our test protocol for ROM, it is possible that a similar artificial drift can explain the systematic bias in our data.

A novel aspect was the measures of ROM in the diagonal plane. Diagonal movements are common functional movements in everyday life and could add value to clinical assessment protocols. The diagonal movements showed mean biases ≤ 1°, which we considered to be very good. However, the diagonal movements had higher LOA (highest diagonal flexion left, ±6.1°), compared to the conventional movement directions (highest lateral flexion left, ±3.4°), which needs to be considered when using the diagonal VR tests. Future research should investigate the diagonal movements for their discriminative validity in people with neck pain, as well as the test–retest reliability to confirm their potential value.

Our results on the concurrent validity of the VR device are in line with previous studies evaluating the validity of VR or other IMU devices for cervical ROM [21,23]. A previous study evaluating cervical ROM using the VR headset Oculus Rift (Irvine CA) found errors of 1.9–4.4° (SD ranging from 4.3 to 10.9°) when compared to the Optotrak Certus 3D camera-based system [21]. This is a larger mean bias than found in our study. The larger standard deviations suggest larger LOA, but this was not analyzed. A head-mounted IMU device for left and right cervical rotation measurements, when tested against the Optotrak Certus [23], also showed high ICC values (0.998), in line with our results. Sarig Bahat et al. (2009) compared VR-based ROM measures performed in a VR game to a conventional measure of ROM using an electromagnetic tracking system (Fastrak, Polhemus). Significant differences were found between the systems. VR gave larger ROM values for the full flexion and extension mean, 7.2° (LOA ± 24.5°), and full rotation mean 16.1° (LOA ± 23.7°). These differences are larger than those found in our study, but Sarig Bahat et al.’s measurements were not collected simultaneously, which could contribute to the differences. Thus, compared to other VR technologies, our VR device seems to be more accurate in the measurements of cervical ROM.

VR technology has advantages over analog clinical assessment tools. The cervical range of motion (CROM) device, while valid [19,40], is less accurate [40] compared to VR measures in our study. The common universal goniometer lacks research on its concurrent validity against a gold-standard measurement system. In addition, the goniometer accuracy depends on clinical experience [41], which is not required with the VR device. Neither the CROM nor the universal goniometer can assess diagonal movements and velocity. Hence, there are several advantages to the VR device.

Our values of the maximum velocities are in line with Röijezon et al.’s study on asymptomatic people [15], but higher than those reported by Sarig Bahat et al. who assessed the peak velocity in a VR game [13]. The instructions for fast rotation were different in the VR game, which may explain these differences. The maximum and mean velocity in our study showed mean biases < 12°/s, which we considered clinically acceptable, although the LOA showed large spreads of differences. The largest errors occurred at maximum velocities above 550°/s, with the VR having about 35–80°/s higher values compared to Qualisys. This indicates that VR is less valid at very high velocities, and caution should be taken if a person move at these high velocities. Our mean value for maximum velocity was 355 (±157) or less. Other studies on asymptomatic people have reported maximum velocities of mean 348°/s (±92) [15] or less [13]. Hence, velocities above 550°/s seem to be rare. They are unlikely to be encountered in patients where the maximum velocity reported is in the vicinity of 226°/s (±88) [15] or less [13,26,42]. The mean velocity had smaller LOA (<±18.7°/s) compared to maximum velocity (<±60°) and might be better for clinical use. However, this needs to be investigated in future studies.

Repeatability is as a measure of precision [39] and our results revealed ICC values > 0.85 for ROM and >0.8 for velocity, indicating the good repeatability of both measures. The absolute mean biases were ≤ 1.3° LOA < ±11.4° for ROM and <32°/s LOA < ±162°/s for velocity. The mean values are clinically acceptable, although the LOA show large spreads. Future research should examine the test–retest reliability over a prolonged time for both VR-assessed ROM and velocity.

The side effects were generally slight and acceptable. One participant felt slightly tired and dizzy for about 10 min after the test session. Side effects such as motion sickness, dizziness, headache [27,43,44], and discomfort [44] have been reported in neck pain VR studies, but no participants experienced these side effects in this study. Thus, there seems to be few side effects from the ROM and velocity tests, but this needs to be monitored in future research.

A strength of this study was the inclusion of participants both with and without neck pain, of both sexes, and with a wide age span. This allowed the concurrent validity to be evaluated with a variety of ROM and velocities. This is important as, in a clinical environment, the ROM and velocity will vary between and within patients. Therefore, there is some generalizability of the results. The VR technology included new diagonal ROM tests and velocity tests which both may have important clinical applications. Future research should investigate the discriminant validity of VR-based cervical movement tests in a larger neck pain cohort.

There are limitations. The study included 20 participants, which is a small sample size, especially for the calculation of limits of agreement. The tests were performed in a controlled laboratory environment with the supervision of a physiotherapist, which might not mimic the clinical situation. This study did not investigate the discriminative validity or responsiveness, which are important aspects to be evaluated in future studies on people with neck pain.

### Clinical Implications

Objective and precise analysis of movements has traditionally involved complex and expensive high-technology measurement systems, often requiring a laboratory setting [32]. This equipment is rarely used in a clinical setting, which stresses the need for new affordable off-the-shelf technology, such as VR and other sensor technology. VR technology offers the automatized calculation of relevant movement variables. VR technology can assess diagonal movements and velocity which other clinical assessment tools cannot do. VR technology can be used for training, where it has the benefit of being a fun type of rehabilitation, which can increase the motivation to perform exercises [45], as well as provide an external focus of attention, which may enhance motor learning [46]. VR technology can also be used in digital health where rehabilitation is supervised and monitored from a distance. Future research is needed to evaluate the effect on neck pain and function of VR-based interventions for patients with neck pain. The usability of VR technology both from a patient and physiotherapist perspective should also be investigated.

## 5. Conclusions

This study demonstrated that VR technology has an acceptable validity for the assessment of the cervical range of motion and velocity. It has good precision in providing instant test results. It can be used to assess functional diagonal cervical movements, which previously was only undertaken in a laboratory setting. This supports the idea of “taking the lab to the clinic” and the use of VR as a “mini lab”. This opens up new possibilities for more tailored rehabilitation, as important movement functions can be assessed and exercised with precision.

## Figures and Tables

**Table 1:** Paired-samples *t*-test ROM variables. VR compared to Qualisys.

Cervical Movement Direction	Qualisys Mean (SD)°	VR Mean (SD)°	Mean Difference°	Std. Deviation	Std. Error of Mean	95% Confidence Interval of the Difference		*t*- Value	*p*
						Lower	Upper		
Rotation right	66.7 (8.5)	64.6 (8.8)	2.1	1.5	0.3	1.4	2.8	6.15	<0.001
Rotation left	70.4 (9.9)	70.9 (9.7)	−0.6	1.6	0.4	−1.3	0.2	−1.59	0.128
Extension	64.7 (10.7)	64.8 (11.0)	−0.1	0.8	0.2	−0.5	0.3	−0.55	0.589
Flexion	48.0 (10.1)	48.2 (10.3)	−0.2	0.7	0.2	−0.6	0.1	−1.30	0.210
Lateral flexion right	39.4 (10.2)	40.0 (9.7)	−0.7	1.6	0.4	−1.4	0.1	−1.77	0.092
Lateral flexion left	39.3 (10.7)	39.6 (11.2)	−0.3	1.7	0.4	−1.1	0.5	−0.75	0.462
Diagonal extension right	64.5 (10.0)	64.7 (10.6)	−0.2	2.7	0.6	−1.4	1.1	−0.28	0.780
Diagonal extension left	67.0 (10.0)	67.5 (10.0)	−0.5	1.5	0.3	−1.2	0.2	−1.45	0.164
Diagonal flexion right	62.3 (10.8)	61.3 (10.9)	1.0	2.6	0.6	−0.3	2.2	1.66	0.113
Diagonal flexion left	62.9 (11.8)	62.1 (11.0)	0.7	3.1	0.7	−0.7	2.2	1.07	0.298

ROM: range of motion; VR: virtual reality; °: degrees; SD: standard deviation; *p*: significance two-sided *p*-value.

**Table 2:** Pearson’s r and ICC (2.1) 95% CI. ROM variables. VR compared to Qualisys.

Cervical Movement Direction	Pearson r	ICC (2.1) 95% CI
Rotation right	0.986 ***	0.958 *** 0.396 to 0.990
Rotation left	0.987 ***	0.986 *** 0.964 to 0.994
Extension	0.997 ***	0.997 *** 0.993 to 0.999
Flexion	0.998 ***	0.997 *** 0.993 to 0.999
Lateral flexion right	0.988 ***	0.985 *** 0.962 to 0.994
Lateral flexion left	0.988 ***	0.988 *** 0.969 to 0.995
Diagonal extension right	0.967 ***	0.967 *** 0.918 to 0.987
Diagonal extension left	0.989 ***	0.988 *** 0.970 to 0.995
Diagonal flexion right	0.971 ***	0.969 *** 0.922 to 0.988
Diagonal flexion left	0.965 ***	0.963 *** 0.910 to 0.985

ICC: Intraclass correlation Coefficient; 2.1: two-way random effects model, absolute agreement, single measures; CI: Confidence Interval; *** *p*-value < 0.001.

**Table 3:** Bland–Altman bias and 95% LOA. ROM variables. VR compared to Qualisys.

Cervical Range of Motion Variable	Mean Bias° (Qualisys Minus VR)	Median Bias° (Qualisys Minus VR)	95% LOA°		LOA Percentiles°	
			Lower	Upper	5th	95th
Rotation right ^1^	2.1	**2.6**	−0.9	5.0	**−1.7**	**4.0**
Rotation left	−0.6	−0.7	−3.7	2.6	−3.1	4.0
Extension	−0.1	−0.2	−1.7	1.5	−1.5	1.4
Flexion ^1^	−0.2	**−0.2**	−1.7	1.2	**−2.1**	**0.7**
Lateral flexion right	−0.7	−0.9	−3.9	2.6	−2.8	2.5
Lateral flexion left	−0.3	−1.0	−3.7	3.1	−3.1	2.7
Diagonal extension right ^1^	−0.2	**0.5**	−5.5	5.1	**−9.2**	**3.3**
Diagonal extension left	−0.5	−0.5	−3.4	2.5	−2.9	1.8
Diagonal flexion right	1.0	0.8	−4.1	6.0	−3.0	7.1
Diagonal flexion left ^1^	0.7	**0.5**	−5.4	6.8	**−4.3**	**10.6**

°: degrees; LOA: Limits of agreement. Negative values represent higher values for VR device, positive values represent lower values for VR. ^1^ = skewed distribution of differences, median, and percentiles (bold values) used for Bland–Altman plots.

**Table 4:** Paired-samples *t*-test. Velocity. VR compared to Qualisys.

	Qualisys Mean (SD)°/s	VR Mean (SD)°/s	Mean Difference °/s	SD	Std. Error Mean	95% Confidence Interval of the Difference		*t*	*p*
						Lower	Upper		
Maximum velocity									
Rotation right	322.7 (135.9)	330.4 (145.3)	−7.7	17.0	3.9	−15.9	0.5	−1.98	0.063
Rotation left	343.4 (134.1)	355.1 (157.2)	−11.7	30.6	7.0	−26.4	3.0	−1.67	0.112
Mean velocity									
Rotation right	180.2 (89.1)	178.7 (85.0)	1.5	9.5	2.2	−3.1	6.1	0.68	0.507
Rotation left	191.4 (92.2)	194.4 (95.9)	−3.0	8.3	1.9	−7.1	1.0	−1.59	0.129

°/s: degrees per second; SD: standard deviation.

**Table 5:** Pearson’s r and ICC (2.1) 95% CI. Maximum and mean velocity. VR compared to Qualisys.

Velocity Variables	Pearson r	ICC (2.1) 95% CI
Maximum velocity		
Rotation right	0.995 ***	0.992 *** 0.976 to 0.997
Rotation left	0.991 ***	0.976 *** 0.938 to 0.991
Mean velocity		
Rotation right	0.995 ***	0.994 *** 0.985 to 0.998
Rotation left	0.997 ***	0.996 *** 0.989 to 0.998

*** *p* < 0.001.

**Table 6:** Bland–Altman bias and limits of agreement. Velocity variables. VR compared to Qualisys.

	Mean Bias°/s (Qualisys Minus VR)	Median Bias°/s (Qualisys Minus VR)	95% LOA°/s		LOA Percentiles °/s	
			Lower	Upper	5th	90th
Maximum velocity						
Rotation right	−7.7	−10.1	−40.9	25.5	−43.7	18.9
Rotation left ^1^	−11.7	**−0.5**	−71.6	48.2	**−83.3**	**18.6**
Mean velocity						
Rotation right	1.5	1.0	−17.2	20.2	−16.2	14.6
Rotation left ^1^	−3.0	**−1.4**	−19.4	13.3	**−30.8**	**4.3**

°/s: degrees per second; LOA: Limits of agreement. Negative values represent higher values for VR device, positive values represent lower values for VR. ^1^ = skewed distribution of differences, median, and percentiles (bold values) used for Bland–Altman plots.

## Data Availability

The data presented in this study are available on request from the corresponding author.

## Appendix A

**Table A1:** Paired-samples *t*-test. Repeatability VR ROM.

	VR Rep1 Mean (SD)°	VR Rep2 Mean (SD)°	Mean Difference °	Std. Deviation	Std. Error of Mean	95% Confidence Interval of the Difference		*t*	Significance Two-Sided *p*
						**Lower**	**Upper**		
Rotation right	64.6 (8.8)	64.5 (10.3)	0.1	3.8	0.8	−1.6	1.9	0.15	0.882
Rotation left	70.9 (9.7)	71.0 (11.4)	−0.1	4.6	1.0	−2.2	2.1	−0.08	0.934
Extension	64.8 (11.0)	64.3 (12.0)	0.4	4.3	1.0	−1.6	2.4	0.44	0.666
Flexion	48.2 (10.3)	48.0 (11.4)	0.2	5.1	1.1	−2.2	2.6	0.16	0.878
Lateral flexion right	40.0 (9.7)	39.4 (9.5)	0.7	3.7	0.8	−1.1	2.4	0.79	0.439
Lateral flexion left	39.6 (11.2)	38.2 (10.9)	1.3	2.6	0.6	0.1	2.5	2.28	0.034 *
Diagonal extension right	64.7 (10.6)	65.9 (10.7)	−1.2	5.8	1.3	−3.9	1.5	−0.95	0.353
Diagonal extension left	67.5 (10.0)	68.3 (9.9)	−0.8	3.4	0.8	−2.3	0.8	−1.01	0.324
Diagonal flexion right	61.3 (10.9)	60.6 (11.7)	0.7	4.3	1.0	−1.3	2.7	0.76	0.454
Diagonal flexion left	62.1 (11.0)	61.5 (12.7)	0.7	4.3	1.0	−1.4	2.7	0.68	0.503

°: degrees; * Significant (*p* < 0.05); t: t-value; VR: virtual reality; ROM: range of motion.

**Table A2:** Pearson’s and ICC (3.1). Repeatability VR ROM.

Cervical Movement Direction	Pearson’s r	ICC (3.1) 95% CI
Rotation right	0.934 ***	0.927 *** 0.824 to 0.970
Rotation left	0.917 ***	0.910 *** 0.787 to 0.963
Extension	0.934 ***	0.933 *** 0.840 to 0.973
Flexion	0.896 ***	0.895 *** 0.754 to 0.957
Lateral flexion right	0.926 ***	0.927 *** 0.827 to 0.970
Lateral flexion left	0.973 ***	0.967 *** 0.906 to 0.988
Diagonal extension right	0.852 ***	0.852 *** 0.669 to 0.938
Diagonal extension left	0.944 ***	0.943 *** 0.865 to 0.977
Diagonal flexion right	0.931 ***	0.930 *** 0.834 to 0.972
Diagonal flexion left	0.944 ***	0.936 *** 0.847 to 0.974

*** *p*-value < 0.001; ICC: intraclass correlation coefficient; ICC 3.1: two-way mixed effects, absolute agreement.

**Table A3:** Bland–Altman bias and 95 % LOA. Repeatability VR ROM.

	Mean Bias° (Rep 1 minus Rep 2)	Median Bias° (Rep 1 minus Rep 2)	95% LOA°		LOA Percentiles°	
			Lower	Upper	5th Percentile	95th Percentile
Rotation right **^1^**	0.1	**0.6**	−7.2	7.5	**−11.0**	**4.5**
Rotation left	−0.1	−0.7	−9.1	8.9	−6.5	9.3
Extension	0.4	−0.6	−8.0	8.8	−6.2	7.6
Flexion	0.2	0.6	−9.8	10.1	−13.9	8.3
Lateral flexion right	0.7	0.2	−6.6	7.9	−5.0	10.3
Lateral flexion left	1.3	1.2	−3.7	6.4	−3.7	6.4
Diagonal extension right	−1.2	−1.8	−12.6	10.1	−9.4	10.4
Diagonal extension left	−0.8	0.1	−7.3	5.8	−7.2	5.0
Diagonal flexion right	0.7	0.1	−7.6	9.1	−7.7	8.9
Diagonal flexion left	0.7	1.2	−7.8	9.1	−8.7	7.0

°: degrees; LOA: limits of agreement. Negative values represent lower value for rep 1, positive value represents higher value for rep 1. **^1^** = skewed distribution of differences, median, and percentiles (bold values) used for Bland–Altman plots.

**Table A4:** Paired-samples *t*-test. Repeatability VR velocity, repetitions 1 and 2.

Velocity Variables	VR Rep 1 Mean (SD) °/s	VR Rep 2 Mean (SD) °/s	Mean Difference (SD) °/s	Std. Error of Mean	95% Confidence Interval of the Difference		*t*	*p*
					Lower	Upper		
Maximum velocity								
Right Rotation	299.9 (141.9)	331.6 (151.8)	−31.7 (82.5)	18.9	−71.5	8.0	−1.68	0.111
Left Rotation	350.8 (154.0)	352.5 (157.6)	−1.7 (52.1)	12.0	−26.8	23.4	−0.14	0.889
Mean velocity								
Right Rotation	161.1 (77.1)	185.3 (96.4)	−24.2 (50.5)	11.6	−48.6	0.1	−2.09	0.051
Left Rotation	191.8 (91.6)	192.6 (95.7)	−0.8 (32.0)	7.3	−16.2	14.6	−0.11	0.915

°/s: degrees per second; SD: standard deviation; *p*: significance two-sided *p*-value.

**Table A5:** Pearson’s r and ICC (3.1). Repeatability VR velocity repetitions 1 and 2.

Velocity Variables	Pearson’s r	ICC (3.1) 95% CI
Maximum Velocity		
Right Rotation	0.844 ***	0.830 *** 0.611 to 0.931
Left Rotation	0.944 ***	0.947 *** 0.867 to 0.979
Mean Velocity		
Right Rotation	0.854 ***	0.809 *** 0.548 to 0.923
Left Rotation	0.943 ***	0.944 *** 0.862 to 0.978

*** = *p*-value < 0.001; ICC: intraclass correlation coefficient; ICC 3.1: two-way mixed effects, absolute agreement.

**Table A6:** Bland–Altman bias and limits of agreement. VR velocity repeatability.

	**Mean** **Bias°/s** **(VR Rep 1 minus VR Rep 2)**	**Median** **Bias°/s** **(VR Rep 1** **minus VR Rep 2)**	**95% LOA°/s**		**LOA Percentiles °/s**	
			**Lower**	**Upper**	**5th**	**90th**
Maximum velocity						
Rotation right **^1^**	−31.7	**−19.5**	−193.3	129.9	**−263.7**	**38.0**
Rotation left	−1.7	−6.0	−103.8	100.4	−109.4	76.3
Mean velocity						
Rotation right **^1^**	−24.2	**−5.7**	−123.2	74.7	**−151.6**	**26.0**
Rotation left	−0.8	−2.4	−63.5	62.0	−62.1	42.0

°/s: degrees per second; LOA: limits of agreement. Negative values represent lower value for rep 1, positive value represents higher value for rep 1. **^1^** = skewed distribution of differences, median, and percentiles (bold values) are used for Bland–Altman plots.
